# TGF-β and Physiological Root Resorption of Deciduous Teeth

**DOI:** 10.3390/ijms18010049

**Published:** 2016-12-27

**Authors:** Emi Shimazaki, Takeo Karakida, Ryuji Yamamoto, Saeko Kobayashi, Makoto Fukae, Yasuo Yamakoshi, Yoshinobu Asada

**Affiliations:** 1 Department of Pediatric Dentistry, School of Dental Medicine, Tsurumi University, 2-1-3 Tsurumi, Tsurumi-ku, Yokohama 230-8501, Japan; shimazaki-emi@tsurumi-u.ac.jp (E.S.); kinoshita-saeko@tsurumi-u.ac.jp (S.K.); asada-y@tsurumi-u.ac.jp (Y.A.); 2 Department of Biochemistry and Molecular Biology, School of Dental Medicine, Tsurumi University, 2-1-3 Tsurumi, Tsurumi-ku, Yokohama 230-8501, Japan; karakida-t@tsurumi-u.ac.jp (T.K.); yamamoto-rj@tsurumi-u.ac.jp (R.Y.); fukae-m@tsurumi-u.ac.jp (M.F.)

**Keywords:** cytokine, gene expression, osteoclast, root resorption, pediatric dentistry, protein expression

## Abstract

The present study was performed to examine how transforming growth factor β (TGF-β) in root-surrounding tissues on deciduous teeth regulates the differentiation induction into odontoclasts during physiological root resorption. We prepared root-surrounding tissues with (R) or without (N) physiological root resorption scraped off at three regions (R1–R3 or N1–N3) from the cervical area to the apical area of the tooth and measured both TGF-β and the tartrate-resistant acid phosphatase (TRAP) activities. The TGF-β activity level was increased in N1–N3, whereas the TRAP activity was increased in R2 and R3. In vitro experiments for the receptor activator of nuclear factor-κB (NF-κB) ligand (RANKL)-mediated osteoclast differentiation revealed that proteins from N1–N3 and R1–R3 enhanced the TRAP activity in RAW264 cells. A genetic study indicated that the mRNA levels of TGF-β1 in N1 and N2 were significantly increased, and corresponded with levels of osteoprotegerin (OPG). In contrast, the expression level of RANKL was increased in R2 and R3. Our findings suggest that TGF-β is closely related to the regulation of OPG induction and RANKL-mediated odontoclast differentiation depending on the timing of RANKL and OPG mRNA expression in the root-surrounding tissues of deciduous teeth during physiological root resorption.

## 1. Introduction

Physiological root resorption of human deciduous teeth proceeds from a root non-resorption period to a root resorption period for the formation of permanent teeth. This series of progressions is mediated by odontoclasts, which are produced in the root-surrounding tissues [1]. Odontoclasts possess similar characteristics, such as the ultrastructure, enzymatic, and metabolic properties, as osteoclasts [2,3,4,5]. During osteoclastogenesis, the receptor activator of nuclear factor-κB (NF-κB) ligand (RANKL) and its receptor (RANK) have been shown to play a crucial role in the induction of osteoclast differentiation [6], whereas osteoprotegerin (OPG) is a decoy receptor for RANKL and suppresses osteoclast differentiation [7]. Thus, this mechanism must be taken into consideration, even in odontoclastogenesis.

In periodontal tissue, osteoblasts in the alveolar bone, periodontal ligament (PDL) cells, cementoblasts, and gingival epithelial cells express RANKL rather than OPG [8,9,10,11]. It has been reported that PDL cells exclusively express OPG under non-resorption conditions, whereas the RANKL expression levels become up-regulated and those of OPG become down-regulated under resorption conditions [12]. Other studies have shown that cytokines released from tooth germ regulate the balance between RANKL and OPG in osteoclasts and osteoclastic bone resorption in alveolar bone [13,14]. Previous experiments by our group have shown that transforming growth factor-β (TGF-β), which is a multifunctional cytokine and is critical for cell differentiation, exists in the pulp and periodontal ligaments [15].

In mammals, TGF-β consists of three isoforms, including TGF-β1, TGF-β2, and TGF-β3, and their structures are highly conserved among isoforms and species [16]. TGF-β1 negatively regulates osteoclastogenesis via OPG induction with bone marrow stromal cells [17]. Furthermore, TGF-β has been shown to induce OPG expression in osteoblastic cells and inhibit osteoclast differentiation and survival [18]. In contrast, TGF-β exerts contradictory actions on osteoclasts via the regulation of both the proliferation and fusion of osteoclast precursors and the enhancement of RANKL-mediated differentiation through various TGF-β signaling pathways [19,20,21,22,23].

Our aim in this study is to characterize the action of TGF-β in tissues that surround deciduous teeth during physiological root resorption at both the protein and mRNA levels.

## 2. Results

### 2.1. Sequential Extraction of Proteins from Root-Surrounding Tissues

**Figure 1:**
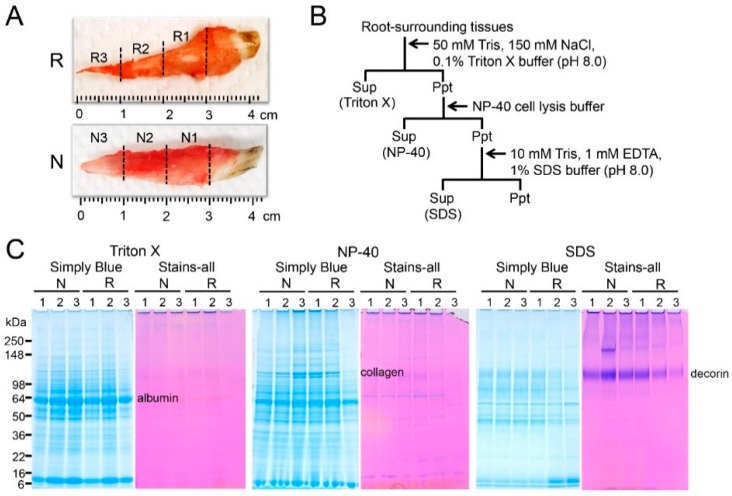
Extraction of proteins in root-surrounding tissues on deciduous incisor teeth. (**A**) Deciduous incisor teeth with (R) or without (N) root resorption from five-month-old pigs. The root-surrounding tissues were scraped off by dividing them into three regions (R1–R3 and N1–N3) at 1-cm intervals from the cervical area to the apical area of the tooth; (**B**) Flow chart indicates the procedures used to produce extracts for proteins in root-surrounding tissues. Sup: supernatant; Ppt: precipitate; (**C**) SDS-PAGE (5% to 20% gradient gel) stained with Simply Blue Safe Stain (Simply Blue) and Stains-all indicates each fraction.

Since TGF-β activity has been found in the pulp and periodontal ligaments [15], we initially attempted to detect in vivo TGF-β in root-surrounding tissues at the protein level. To achieve this purpose, we performed the sequential extraction. We dissected deciduous teeth with or without root resorption from five-month-old pigs and scraped off the surrounding tissues on three regions (R1–R3 and N1–N3) at one-centimeter intervals from the cervical areas to the apical area of the tooth (Figure 1A). The scraped surrounding tissues were submitted to a series of three extractions with Triton X, NP-40, and SDS (sodium dodecyl sulfate) buffers (Figure 1B). These extractions yielded three soluble fractions, designated as Triton X, NP-40, and SDS fractions, which were analyzed via SDS-PAGE (polyacrylamide gel electrophoresis) stained with Simply Blue Safe Stain and Stains-all (Figure 1C). In all fractions, R3 obtained from the apical area with root resorption was prone to reduce protein bands. The albumin and collagen were mainly collected in the Triton X and NP-40 fractions, respectively. Interestingly, the collagen bands in both the Triton X and NP-40 fractions obtained from R3 were substantially reduced compared with the other fractions; however, the intensity of the albumin band was minimally changed. The Stains-all positive decorin band had an apparent molecular weight of 110 kDa in the SDS fraction, which was reduced in the following order in the deciduous teeth with root resorption: R1 > R2 > R3.

### 2.2. Detection of TGF-β and Tartrate-Resistant Acid Phosphatase (TRAP) Activities in Root-Surrounding Tissues and Identification of TGF-β Isoforms

**Figure 2:**
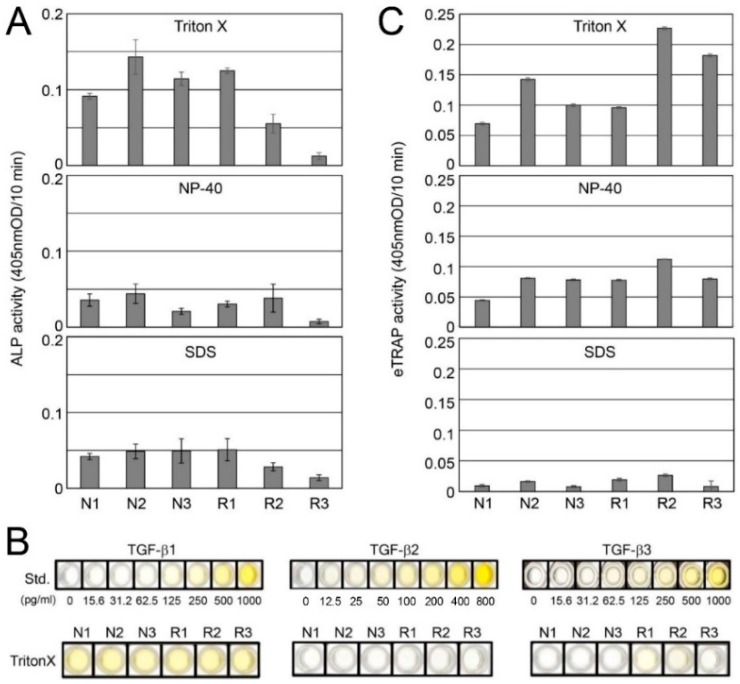
Detection of alkaline phosphatase (ALP)-inducing activities and endogenous TRAP (eTRAP) activities in root-surrounding tissues, and identification of TGF-β isoforms. (**A**) ALP-inducing activity of HPDL cells exposed by protein extracts in N1–N3 and R1–R3 extracted with Triton X, NP-40, and SDS buffers from root surrounding tissues (*n* = 6 culture wells per sample); (**B**) enzyme-linked immunosorbent assay (ELISA) for the detection of TGF-β1, TGF-β2, and TGF-β3 in N1–N3 and R1–R3 extracted with Triton X; (**C**) eTRAP activity in N1–N3 and R1–R3 extracted with Triton X, NP-40, and SDS buffers from root surrounding tissues (*n* = 3 tests per sample).

We determined the alkaline phosphatase (ALP)-inducing activity in human periodontal ligament (HPDL) cells enhanced by TGF-β in the Triton X, NP-40, and SDS fractions. The ALP-inducing activity among the three fractions was high in the following order: Triton X > SDS > NP-40 (Figure 2A). In the Triton X fraction, the ALP activity was overall high at all regions in the N group; however, its activity in the R group was reduced in association with root resorption (R1 > R2 > R3). The ALP activity in both the NP-40 and SDS fractions was decreased compared with Triton X. A trace level of ALP activity was detected in all R3 samples. In the Triton X fraction, the enzyme-linked immunosorbent assay (ELISA) against TGF-β1 antibody showed a detectable signal within the range of 25 to 100 pg per one mg of sample; however, the assay against TGF-β2 antibody showed trace levels of detection. The ELISA against TGF-β3 antibody weakly showed a positive detection in both R1 and R2 within the range of 3.12 to 12.5 pg per one mg of sample; however, it was minimally detected in the other samples (Figure 2B).

To gain information regarding odontoclasts in each sample, we examined the endogenous TRAP (eTRAP) activity in the Triton X, NP-40, and SDS fractions. The eTRAP activity among the three fractions was high in the following order: Triton X > NP-40 > SDS fractions. In the Triton X fraction, the eTRAP activity in both R2 and R3 were approximately 2.3–3.8-fold higher than R1 (Figure 2C).

### 2.3. In Vitro Experiment for RANKL-Mediated Osteoclast Differentiation

**Figure 3:**
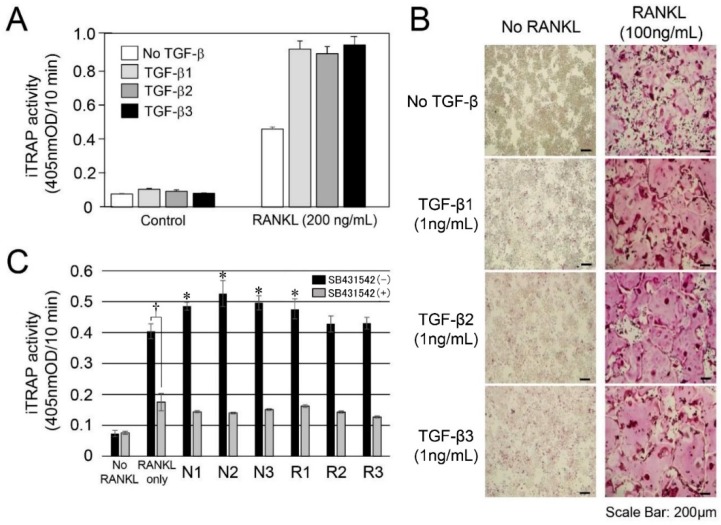
In vitro experiment for RANKL (receptor activator of NF-κB ligand)-mediated osteoclast differentiation. (**A**) Induced TRAP (iTRAP) activity of RAW264 cells exposed by TGF-β isoforms (1 ng/mL each) with (RANKL) or without (Control) the addition of RANKL (200 ng/mL) (*n* = 6 culture wells per group); (**B**) TRAP staining for RAW264 cells exposed by TGF-β isoforms without (**left** panel) or with (**right** panel) RANKL (100 ng/mL); (**C**) iTRAP activity of RAW264 cells exposed by protein extracts in N1–N3 and R1–R3 of root surrounding tissues with or without the addition of GST (glutathione *S*-transferase)-RANKL (200 ng/mL) and SB431542 (1 µM). In the RANKL only sample, the dagger (†) indicates a significant difference between with and without SB431542. The asterisk (*) on the bar graph without SB431542 indicates a significant difference between each sample and RANKL only. In the group with SB431542, there were no significant differences between each sample and RANKL only. Statistical significance (* or †) was determined with an unpaired Student’s *t*-tests. In all cases, *p* < 0.05 was regarded as statistically significant.

**Figure A1:**
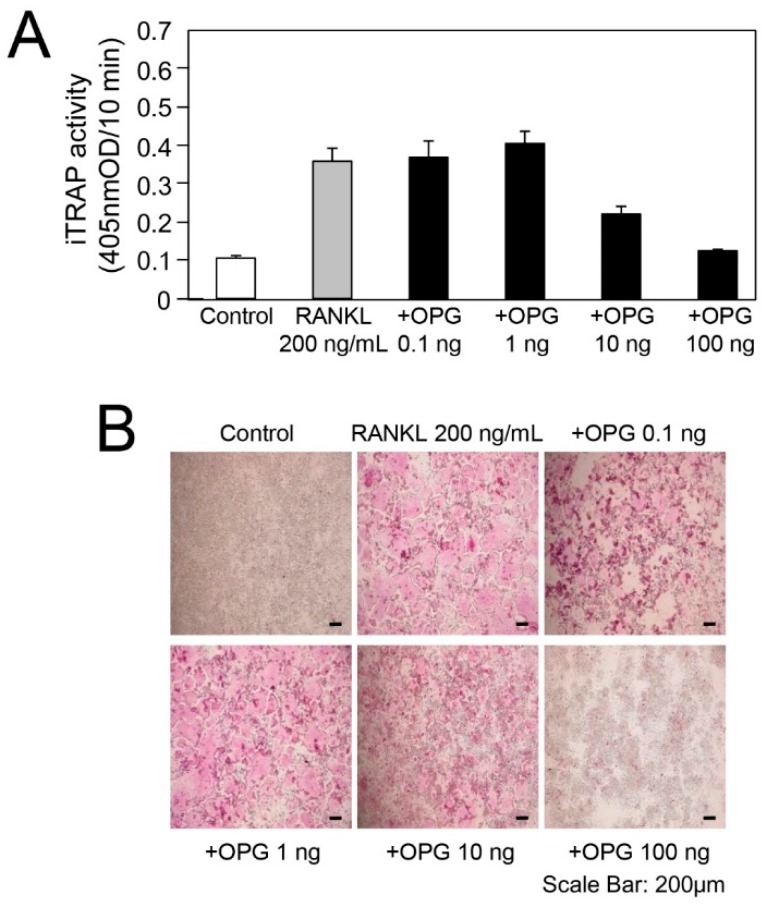
Effect of OPG against RANKL stimulated RAW264 cells. (**A**) iTRAP activity (*n* = 6 culture wells for each group) and (**B**) TRAP staining of RAW264 cells exposed by RANKL only (200 ng/mL) without (Control) or with the addition of various concentration of OPG (0.1 to 100 ng).

We confirmed the TGF-β activity in the protein extracts from root-surrounding tissues; thus, we further attempted to perform in vitro odontoclast differentiation experiments. In this experiment, we used RAW264 cells, which have been widely used for osteoclast differentiation experiments because odontoclasts possess similar characteristics to osteoclasts [2,3,4,5]. We initially confirmed that three recombinant human TGF-βs (rhTGF-β1, rhTGF-β2, and rhTGF-β3) evenly induced RANKL-mediated osteoclast differentiation (Figure 3A), and the addition of OPG suppressed it (Figure A1: Effect of OPG against RANKL stimulated RAW264 cells). The findings regarding TRAP staining for osteoclasts were compared with the controls without RANKL. The addition of each TGF-β isoform enhanced the dyeability of TRAP staining (Figure 3B).

TGF-β activity was highly detected in the Triton X fraction (Figure 2A); thus, we used it for the osteoclast differentiation experiments. The induced TRAP (iTRAP) activity in the RANKL-stimulated RAW264 cells without the addition of N1–N3 and R1–R3 of the Triton X fraction was increased approximately 5.0-fold compared with the RANKL-unstimulated RAW264 cells. The addition of each Triton X fraction enhanced the iTRAP activity level in the N group (approximately 1.2–1.3-fold) and the R group (approximately 1.08–1.1-fold) against the level of RANKL only as a baseline (Figure 3C). Interestingly, the level of increased iTRAP activity nearly corresponded to the ALP-inducing activity in the Triton X fraction, as shown in Figure 2A. When we cultured N1-N3 and R1-R3 with SB431542, the iTRAP activity level was almost equal with that of RANKL only with SB431542, which is a specific and selective inhibitor of TGF-β type I activin receptor-like kinase (ALK) receptors, as a baseline.

### 2.4. Gene Expression in Root-Surrounding Tissues

**Figure 4:**
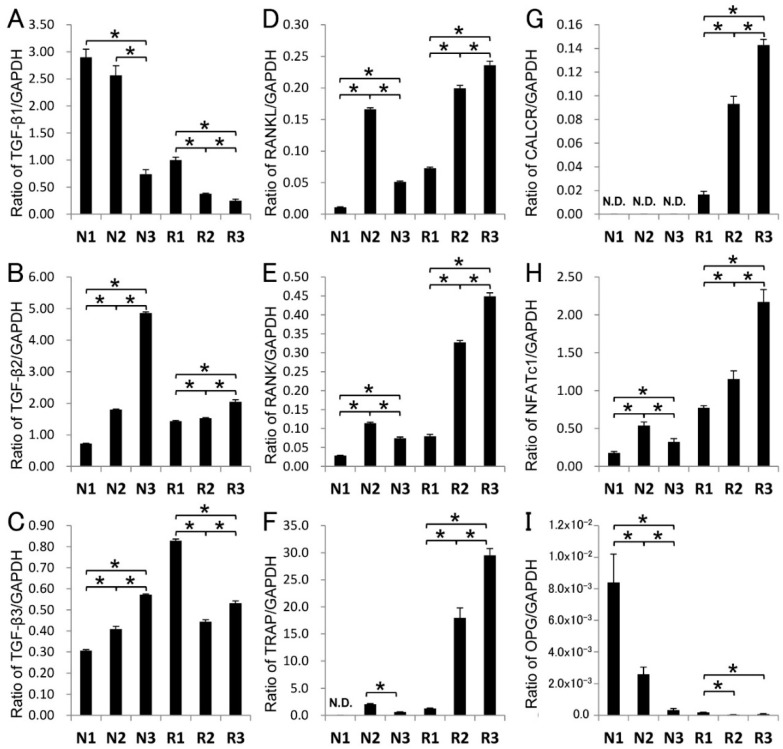
Gene expression in root surrounding tissues. mRNA expressions via qPCR analysis of (**A**) TGF-β1, (**B**) TGF-β2, (**C**) TGF-β3, (**D**) RANKL, (**E**) RANK, (**F**) TRAP, (**G**) CALCR, (**H**) NFATc1 and (**I**) OPG were shown. Each ratio was normalized to the relative quantification data of each mRNA in N1–N3 and R1–R3 of root-surrounding tissues compared with a reference gene (GAPDH), which was generated on the basis of a mathematical model for relative quantification in a qPCR system (*n* = 6 tissues). Statistical significance (*) was determined with an unpaired Student’s *t*-tests. In all cases, *p* < 0.05 was regarded as statistically significant.

We identified all TGF-β isoforms, although TGF-β2 was the trace level in the Triton X fraction (Figure 2B); thus, we further attempted to investigate the genetic information of odontoclast differentiation in root-surrounding tissues. We performed quantitative real-time PCR (qPCR) assays with TGF-β1, TGF-β2, TGF-β3, RANKL, RANK, TRAP/ACP5, calcitonin receptor (CALCR), and nuclear factor of activated T-cells 1 (NFATc1) representing odontoclast promotion and OPG representing odontoclast suppression (Figure 4). To obtain quantitative information, we normalized the quantity of the root-surrounding tissue per sample in each amplification with GAPDH (glyceraldehyde-3-phosphate dehydrogenase) using a standard mathematical model for relative quantification in a qPCR system; this approach assumed a comparable proportion of odontoclasts in each sample. The expression levels of TGF-β1 mRNA in N1 and N2 were increased (approximately 3.4–3.9-fold) compared with N3, whereas the TGF-β1 mRNA level in R1 was increased (approximately 2.5–3.3-fold) compared with R2 and R3. The mRNA level of both TGF-β2 and TGF-β3 in the N group exhibited the opposite of the pattern and the TGF-β1. Compared with N1 and N2, the mRNA level in N3 of TGF-β2 and TGF-β3 was increased approximately 2.6–6.0-fold and 1.5–2.0-fold, respectively.

The mRNA levels of RANKL in R2 and R3 were increased (approximately 2.9–3.4-fold) compared with R1, and the level increased with the progression of root resorption. The expression pattern of RANKL mRNA in the R group was approximately the same as the other osteoclast marker genes, such as RANK, TRAP, CALCR, and NFATc1. The RANKL mRNA level in N2 was also increased compared with N1 and N3 (approximately 3.5–8.8-fold). The mRNA levels of OPG in N1 and N2 were significantly increased (approximately 12–40-fold) compared with N3. Only a trace expression of OPG was identified in the R group. Based on our qPCR analysis, we calculated the RANKL/OPG ratio; the ratio of N1:N2:N3 was 1.34:63.8:157, whereas the ratio R1:R2:R3 was 393:5192:3092.

We summarized the relationships of TGF-β, protein components, and odontoclastogenesis-associated factors in the root-surrounding tissues of deciduous teeth during the physiological root resorption in Table 1.

## 3. Discussion

### 3.1. TGF-β in Root-Surrounding Tissues of Deciduous Teeth

We used deciduous incisor teeth from five-month-old pigs with or without root resorption (six teeth for each group). At this stage, all the teeth were approximately 4 centimeters long. We have previously shown that TGF-β1 binds to both amelogenin, major enamel protein and dentin sialophosphoprotein (DSPP)-derived proteins, most abundant dentin non-collagenous proteins. The amounts of TGF-β1 bound to amelogenin and DSPP-derived proteins were approximately 200–800 pg/mg of amelogenin and 270–380 pg/mg of DSPP-derived proteins, respectively [24,25]. For those studies, we used ten µg of each protein sample (i.e., 2–8 pg of TGF-β1) for detecting ALP-inducing activity (i.e., TGF-β activity) by using ALP-HPDL system. Therefore, we decided to divide each root into three regions (R1–R3 and N1–N3) by an interval of one-centimeter to obtain a viable amount of TGF-β not only for ALP-HPDL system, but also for in vitro RANKL-mediated osteoclast differentiation experiment. Based on this idea, we were able to detect a signal within the range of 25 to 100 pg of TGF-β1 and 3.12 to 12.5 pg of TGF-β3 per one mg of sample by ELISA, respectively.

Decorin binds to TGF-β and represents the candidate for the sequestration of TGF-β reservoirs [26,27,28,29]. Decorin contains independent binding sites for TGF-β and type I collagen [30]. We demonstrated that TGF-β, collagen, and decorin were independently extracted in the Triton X, NP-40, and SDS fractions, and the SDS-PAGE patterns of both collagen and decorin were reduced with the progression of root resorption (i.e., R1 > R2 > R3). Based on the finding that the ALP-inducing activity in the Triton X fraction also decreased with the progression of root resorption, our data suggest that both collagen and decorin provide a retention capability for TGF-β activity; however, TGF-β may have dissociated from them during extraction.

### 3.2. TGF-β Activity in Root-Surrounding Tissues of Deciduous Teeth and In Vitro Experiment for RANKL-Mediated Osteoclast Differentiation

In mammals, three TGF-β isoforms (TGF-β1 to -β3) have been identified and their structures are highly conserved among isoforms and species, with each isoform exhibiting similar functions in vitro [31,32]. Our protein study revealed that TGF-β isoforms in root-surrounding tissues of deciduous teeth were mainly TGF-β1 with low amounts of TGF-β3. However, we demonstrated that TGF-β activity of R2 and R3 were lower compared with those of N2 and N3. Considering that root resorptions occurred only in the R group (not in the N group), our results suggest that TGF-β1 and TGF-β3 may not mainly affect RANKL-mediated osteoclast differentiation in R2 and R3 in vivo. Moreover, although we were able to detect the mRNA expression of TGF-β2 in N3, its amount at the protein level was trace in the Triton X fraction. The reason for this contradiction is unclear at present, although we were able to presume protein degradation, existence in another fraction (i.e., NP-40 and/or SDS fractions), or some other physiological regulations.

A recent study has shown that treatment with adrenaline enhanced the mRNA expression of only TGF-β3 and not TGF-β1 or TGF-β2 in RAW264 cells [33]. This isoform-specific regulation of TGF-β mRNA expression in RAW264 cells led us to consider the effects of the three TGF-β isoforms when RAW264 cells were implemented in the present study. Therefore, we initially confirmed that three rhTGF-βs (rhTGF-β1, rhTGF-β2, and rhTGF-β3) evenly induced RANKL-mediated osteoclast differentiation, and the addition of OPG suppressed it. Based on these findings, our in vitro experiments for RANKL-mediated osteoclast differentiation indicated that the RANKL only sample significantly enhanced the iTRAP activity compared with control (i.e., No RANKL) and the addition of N1–N3 and R1–R3 samples of the Triton X fraction into RAW264 cells further raised the iTRAP activity.

SB431542 is a specific and selective inhibitor of TGF-β type I activin receptor-like kinase (ALK) receptors and inhibits the phosphorylation of immobilized Smad3 [34,35]. We demonstrated that the RANKL only sample with SB431542 significantly reduced the iTRAP activity compared with that without SB431542. This means that the endogenous TGF-β activity was suppressed by SB431542. We furthermore demonstrated that the addition of N1–N3 and R1–R3 samples with SB431542 completely suppressed the iTRAP activity until the level of RANKL only with SB431542 was the same as the baseline. These findings suggest that TGF-β was the main cytokine in N1–N3 and R1–R3 samples of the Triton X fraction, and it was associated with RANKL-mediated osteoclast differentiation in vitro. However, iTRAP activities of R2 and R3 were lower compared with those of N2 and N3. This finding supports that TGF-βs may not mainly affect the RANKL-mediated osteoclast differentiation in R2 and R3 in vivo described above. Considering the root resorption in R group, our finding suggests that other cytokines may have been present in NP-40 and/or SDS fractions, and they may have affected RANKL-mediated osteoclast differentiation in R2 and R3 in vivo.

### 3.3. Potential Roles of TGF-β in Root-Surrounding Tissues of Deciduous Teeth

In periodontal tissue, RANKL has been expressed in not only osteoblasts in the alveolar bone but also periodontal ligament cells, cementoblasts, and gingival epithelial cells [8,9,10]. To enhance RANKL-mediated osteoclast differentiation, TGF-β induces RANKL mRNA expression and activates RANKL promoter activity [36,37]. In the root resorption period, we revealed that the mRNA level of RANKL was high in R2 and R3, whereas the mRNA level of TGF-β1 and TGF-β3 and the ALP-inducing activity in the Triton X fraction were high in R1. We also demonstrated that the mRNA levels of osteoclast marker genes, such as RANK, CALCR, TRAP, and NFATc1, were substantially increased in R2 and R3, as well as the eTRAP activity in the Triton X fraction.

During the root resorption period, cytokines other than TGF-βs, such as interleukin-1, interleukin-33, and parathyroid hormone-related peptide, released from the tooth germ have been associated with the promotion of RANKL expression [13,14,38]. Despite the high levels of RANKL mRNA expression in R2 and R3 (approximately 8.0- to 13-fold higher than R1 as the RANKL/OPG ratio), we revealed that the mRNA levels of TGF-β1 and TGF-β3 exhibited a decreasing tendency in R2 and R3, as well as the ALP-inducing activity in the Triton X fraction. Our study of the genetic level also suggests that TGF-βs may not be the main factor; in contrast, other cytokines or bone resorption factors may enhance RANKL mRNA expression.

In addition to RANKL-mediated osteoclast differentiation, TGF-β has been shown to induce OPG mRNA expression in osteoblastic cells and inhibit osteoclast differentiation; however, the effect of TGF-β1 on the expression of OPG mRNA was transient [17,18]. In a molecular basis study, TGF-β directly affected the transcriptional activity of the *OPG* gene, which generates the reciprocal up-regulation of OPG mRNA and the down-regulation of RANKL mRNA [11]. In the root non-resorption period, we demonstrated that the mRNA levels of both TGF-β1 and OPG were significantly increased in N1; however, the level of ALP-inducing activity in the Triton X fraction was decreased compared with N2 and N3. These findings mean that the amount of TGF-β1 as an active protein was small in N1, although its mRNA level was high. Considering that the mRNA of OPG was increased in N1, our findings suggest that other substances in the N1 sample rather than TGF-β1 may dominantly induce OPG mRNA expression.

We also demonstrated that the mRNA levels of RANKL and TGF-β1 were significantly increased in N2. In addition, the ALP-inducing activity in the Triton X fraction indicated it was at the highest level of all samples. Moreover, we revealed that the mRNA levels of RANK and TRAP exhibited an increased tendency in N2; however, the eTRAP activity was approximately equal between N1 and N2 in the Triton X fraction. Considering that the OPG mRNA expression was identified in N2 and the RANKL/OPG ratio in N2 increased approximately 48-fold compared with N1, our data suggest that TGF-β1 may regulate RANKL-mediated osteoclast differentiation in N2; however, the OPG protein in N1 and N2 may suppress RANKL-mediated osteoclast differentiation. The mRNA levels of TGF-β1, OPG, RANK, RANKL, and TRAP were decreased in N3; however, the expression level of TGF-β3 mRNA indicated an increasing tendency. Moreover, the ALP-inducing activity in the Triton X fraction was retained. We demonstrated that the RANKL/OPG ratio in N3 was approximately 2.5-fold higher than N2, and the eTRAP activity in the Triton X fraction in N3 was slightly increased compared with N1 and N2. These findings suggest that the stage of N3 may gradually switch from OPG mRNA expression to RANKL-mediated osteoclast differentiation.

The RANKL/OPG ratio has been used as an indicator of bone resorption, and it represents an important determinant of bone mass and skeletal integrity, such as in fracture healing [39]. We demonstrated that the RANKL/OPG ratio increased with the progression of root resorption (i.e., N1 < N2 < N3 and R1 < R2 < R3). This finding suggests that OPG is transiently expressed during the root non-resorption period, whereas RANKL is rapidly expressed with the progression of root resorption.

The present study demonstrated that TGF-β is closely related to the regulation of both OPG induction and RANKL-mediated osteoclast differentiation, depending on the timing of the mRNA expression of RANKL and OPG factors in the root-surrounding tissues of deciduous teeth during physiological root resorption. Studies are required to perform further experiments such as morphological, histological, and genetic engineering to gain direct evidences. Based on those forms of evidence, studies are further required to identify the inducing substances for regulating and switching OPG/RANKL balance.

## 4. Materials and Methods

All experimental procedures that involved the use of animals were reviewed and approved by the Ethics Committee of the Institute of Tsurumi University, Yokohama, Japan (Project identification code #1318, 1 December 2015).

### 4.1. Tissue Preparation

Tooth germs of deciduous incisor teeth were surgically extracted from the mandibles of five-month-old pigs (six pigs) obtained from the Meat Market of Metropolitan Central Wholesale Market (Tokyo, Japan). Deciduous teeth with (R) or without (N) root resorption (six teeth for each group) were surgically extracted and the surrounding tissues were scraped off at one-centimeter intervals from three regions (R1–R3; approximately 103–157 mg each and N1–N3; approximately 99–109 mg each) from each tooth’s cervical area to the apical area (Figure 1A). Each sample was utilized immediately for RNA isolation or after storage at −80 °C for protein extraction.

### 4.2. Sequential Extraction of Proteins from Root-Surrounding Tissues

The sequential extraction of proteins from the R1–R3 and N1–N3 samples was conducted in the following order: 50 mM Tris-HCl, 150 mM NaCl, 0.1% Triton X buffer (pH 8.0) (Triton X), NP-40 cell lysis buffer (Thermo Fisher Scientific., Waltham, MA, USA) (NP-40) and 10 mM Tris-HCl, 1 mM EDTA, and 1% SDS buffer (pH 8.0) (SDS). The collected tissue weights from each group, the total micrograms of protein per sample, and the micrograms of protein extracted per milligram of tissue are provided in Table A1: Collected tissue weight, raw data from 660 nm protein assay, the total micrograms of protein in each sample, and the micrograms of protein extracted per milligrams of tissue.

### 4.3. Alkaline Phosphatase (ALP) Activity Assay (ALP-HPDL System)

Each sample (10 µg) fractionated with Triton X, NP-40 and SDS was added into human periodontal ligament fibroblast (HPDL) cells (LONZA, Walkersville, MD, USA), and the cell culture and ALP activity were assessed using our previous method [40].

### 4.4. SDS-PAGE

SDS-PAGE was performed with 5% to 20% e-PAGEL mini gel (ATTO Corporation, Tokyo, Japan). The gels were stained with Simply Blue Safe Stain (Life Technologies/Invitrogen, Carlsbad, CA, USA) or Stains-all (Sigma-Aldrich, St. Louis, MO, USA).

### 4.5. Endogenous and Induced Tartrate-Resistant Acid Phosphatase (TRAP) Assays and TRAP Staining

For the endogenous TRAP assay, each sample (10 µg) in Triton X, NP-40, and SDS fractions was directly incubated with 0.1 M *p*-nitrophenylphosphate as a substrate in TRAP buffer. The induced TRAP assay and TRAP staining were performed using our previous method [41]. Each sample (10 µg) in Triton X was added to RAW264 cells with or without GST (glutathione *S*-transferase)-RANKL (200 ng/mL) and SB431542 (1 µM) (Tocris Bioscience, Bristol, UK).

### 4.6. Enzyme-Linked Immunosorbent Assay (ELISA)

The identification of the TGF-β isoforms in the N1–N3 and R1–R3 samples was determined by a sandwich enzyme immunoassay method using a Quantikine ELISA kit (R&D Systems, Inc., Minneapolis, MN, USA) for TGF-β1 and TGF-β2 and an ELISA Cloud-Immunoassay kit (Cloud-Clone Corp., Houston, TX, USA) for TGF-β3. The ELISA for N1–N3 and R1–R3 in the Triton X fraction (4 µg each) was performed following the manufacturer’s instructions.

### 4.7. Quantitative Real-Time PCR (qPCR)

The root-surrounding tissues at the R1–R3 and N1–N3 regions were extracted with RNA extraction reagent (Isogen, Nippon Gene Co., Ltd., Tokyo, Japan). Purified total RNA (2 µg) was reverse transcribed, and qPCR was subsequently performed using the SYBR Green technique on a LightCycler Nano (Roche Diagnostics, Mannheim, Germany). The selected primers, running conditions, and sizes of the amplified product are provided in Table A2: Selected primers, size of amplified product, and running conditions for qPCR analysis. Each ratio was normalized to the relative quantification data of TGF-β1, TGF-β3, RANKL, RANK, TRAP, calcitonin receptor (CALCR), nuclear factor of activated T-cells 1 (NFATc1), and OPG compared with a reference gene (GAPDH), which was generated on the basis of a mathematical model for relative quantification in the qPCR system.

### 4.8. Statistical Analysis

For the qPCR analysis, in an enzyme assay with the ALP-HPDL system and TRAP assay, all values were represented as the means ± standard error (S.E.M.). Statistical significance (* or †) was determined using unpaired Student’s *t*-tests. In all cases, *p* < 0.05 was regarded as statistically significant.

## Figures and Tables

**Table 1:** Relationships among TGF-βs, protein components, and odontoclastogenesis-associated factors in the root-surrounding tissues of deciduous teeth during physiological root resorption.

Root-Surrounding Tissue		Non-Resorption			Resorption		
		Cervical	Center	Apical	Cervical	Center	Apical
Region		N1	N2	N3	R1	R2	R3
Protein study	Collagen	↑	↑↑	↑↑↑	↑↑↑	↑↑	trace
	Decorin	↑	↑↑↑	↑↑	↑↑	↑	trace
	ALP activity	↑	↑↑	↑↑	↑↑	↑	trace
	eTRAP activity	↑	↑	↑↑	↑↑	↑↑↑↑	↑↑↑
In vitro study	iTRAP activity	↑	↑↑	↑	↑	trace	trace
Genetic study (mRNA level)	TGF-β1	↑↑↑↑	↑↑↑↑	↑↑	↑↑	↑	↑
	TGF-β2	↑	↑↑	↑↑↑↑	↑	↑	↑↑
	TGF-β3	↑	↑↑	↑↑↑	↑↑↑	↑	↑↑
	RANKL	trace	↑↑↑	↑	↑	↑↑↑	↑↑↑↑
	RANK	trace	↑↑	↑	↑	↑↑↑	↑↑↑↑
	TRAP	N.D	↑	trace	↑	↑↑↑	↑↑↑↑
	CALCR	N.D	N.D	N.D	↑	↑↑↑	↑↑↑↑
	NFATc1	trace	↑↑	↑	↑↑	↑↑↑	↑↑↑↑
	OPG	↑↑↑↑	↑↑	trace	trace	trace	trace
	RANKL/OPG ratio	1.34	63.8	157	393	5192	3092

N.D: not determined. Arrow (↑) indicates the intensity obtained in each experimental results (↑: low, ↑↑: middle, ↑↑↑: high, ↑↑↑↑: highest).

## Appendix A

A previous study determined that TGF-β activity has been found in the pulp and periodontal ligaments [1]. Because of the denaturing reagent that was used for that study, we modified the extraction procedure for TGF-β by using a combination of non-ionic detergent, such as Triton X and NP-40, and subsequent ionic detergent SDS in the present study. The amount of total protein in each sample was quantitatively determined with Pierce 660 nm protein assay kit (Thermo Fisher Scientific, Waltham, MA, USA). The collected tissue weights from each group, the total micrograms of protein in each sample, and the micrograms of protein extracted per milligrams of tissue are provided in Table A1. The amount of protein was normalized for each sample on total milligrams of tissue basis and was applied per lane for SDS-PAGE (Figure 1C).

The quantitative real-time PCR (qPCR) was performed using the SYBR Green technique on a LightCycler Nano (Roche Diagnostics) against purified total RNA prepared from the root-surrounding tissues at R1-R3 and N1-N3 regions. The selected primers, running conditions, and size of amplified product are provided in Table A2. Each ratio was normalized relative to the quantification data of TGF-β1, TGF-β3, RANKL, RANK, TRAP, CALCR, NFATc1, and OPG in comparison to a reference gene (GAPDH), which was generated on the basis of a mathematical model for relative quantification in qPCR system.

Odontoclasts possess similar characteristics to osteoclasts, such as the ultrastructure, and enzymatic and metabolic properties [2,3,4,5]. RAW264 cells have been widely used for osteoclast differentiation experiments. Therefore, we used RAW264 cells in the present study and considered the differentiation toward osteoclasts as that toward odontoclasts. We tested to see whether RANKL stimulated RAW264 cells have the ability to induce the osteoclast by three TGF-β isoforms; TGF-β1, TGF-β2, and TGF-β3 (Figure 4A,B). To support this test, we also examined the induced TRAP (iTRAP) activity for RANKL stimulated cells with OPG. The iTRAP activity in cells was observed to decrease in a concentration-dependent manner based on OPG (Figure A1). The findings of TRAP staining for osteoclasts were compared with those of controls without OPG. The level of TRAP staining of osteoclasts containing OPG decreased in a concentration-dependent manner based on OPG (Figure A1). Thus, we confirmed that three recombinant human TGF-βs isoforms evenly induced the RANKL-mediated osteoclast differentiation and that the addition of OPG suppressed it. Then, we used these RAW264 cells for proving that in vivo TGF-βs in Triton X fraction induce RANKL-mediated osteoclast differentiation (Figure 3C).

**Table A1:** Collected tissue weight, raw data from 660 nm protein assay, the total micrograms of protein in each sample, and the micrograms of protein extracted per milligrams of tissue.

Triton X	Tissue (mg)	660 Assay (μg/mL)	Total (μg/μL)	μg/mg Tissue
N1	106.3	5450	2779.5/510	26.1
N2	99.45	4700	3543.8/754	35.6
N3	109.2	6500	4680/720	42.9
R1	109.36	3875	2828.8/730	25.9
R2	102.8	6125	3711.8/606	36.1
R3	156.7	5450	2550.6/468	16.3
NP40	Tissue (mg)	660 Assay (μg/mL)	Total (μg/µL)	μg/mg Tissue
N1	106.3	1210	750.2/620	7.06
N2	99.45	1330	771.4/580	7.76
N3	109.2	1630	717.2/440	6.57
R1	109.36	1200	648/540	5.93
R2	102.8	1190	747.3/628	7.27
R3	156.7	960	604.8/630	3.86
SDS	Tissue (mg)	660 Assay (μg/mL)	Total (μg/μL)	μg/mg Tissue
N1	106.3	135	82.4/610	0.78
N2	99.45	120	70.8/590	0.71
N3	109.2	100	56.2/562	0.51
R1	109.36	135	79.7/590	0.73
R2	102.8	145	67.9/468	0.66
R3	156.7	85	39.3/462	0.25

**Table A2:** Selected primers, size of amplified product, and running conditions for qPCR analysis.

Gene		Sequence (5′–3′)	Size (bp)	qPCR Protocol (45 Cycles)					
				Denaturation		Annealing		Extension	
				°C	Second	°C	Second	°C	Second
*TGF-β1*	Forward	TTCATGAACCCAAGGGCTACC	104	95	10	65	10	72	15
	Reverse	CTGGTTGTACAGAGCCAGGAC							
*TGF-β2*	Forward	CAGTGGGAAGACCCCACATC	150			60			
	Reverse	ATGTAAAGTGGACGCAGGCA							
*TGF-β3*	Forward	CTGTGCGTGAATGGCTCTTG	93						
	Reverse	TATCCCCGTTGGGCTGAAAG							
*RANKL*	Forward	ACGATCAACGCCACAGACAT	89			65			
	Reverse	TTGGAGATCTTGGCCCAACC							
*RANK*	Forward	GAAGCACGTCATGGGACTGA	95			60			
	Reverse	CTGGGCAAGTAAGTCTGGGG							
*TRAP*	Forward	AACGTCTCGGCACAGATAGC	101			65			
	Reverse	GACACATTGGACCGTGGGAT							
*CALCR*	Forward	CGCCTGTGGTGGTATCATGT	142						
	Reverse	CAGGGCCGTGGATGATGTAA							
*NFATc1*	Forward	TCGTGGAGAAAGCACCAGAC	91						
	Reverse	TCGACCACCAAGGAATTCGG							
*OPG*	Forward	TGTTCTGGAAACAGTGAATCGAC	80						
	Reverse	ACAGCAAACCTGAAGAACGC							
*GAPDH*	Forward	CCATCACCATCTTCCAGGAG	346						
	Reverse	ACAGTCTTCTGGGTGGCAGT							

## Abbreviations

SDS	Sodium dodecyl sulfate
PAGE	Polyacrylamide gel electrophoresis
NP-40	Nonidet P-40
kDa	Killodalton
ACP5	Acid phosphatase 5
PCR	Polymerase chain reaction
GAPDH	Glyceraldehyde-3-phosphate dehydrogenase
GST	Glutathione *S*-transferase
